# Mutation Bias, rather than Binding Preference, Underlies the Nucleosome-Associated G+C% Variation in Eukaryotes

**DOI:** 10.1093/gbe/evv053

**Published:** 2015-03-18

**Authors:** Ke Xing, Xionglei He

**Affiliations:** ^1^State Key Laboratory of Biocontrol, College of Ecology and Evolution, Sun Yat-sen University, Guangzhou, China; ^2^Guangdong Key Laboratory of Plant Resources, School of Life Sciences, Sun Yat-Sen University, Guangzhou, China

**Keywords:** G+C% variation, nucleosome, mutation

## Abstract

The effects of genetic content on epigenetic status have been extensively studied, but how epigenetic status affects genetic content is not well understood. As a key epigenetic factor the nucleosome structure is highly correlated with local G+C% in eukaryotic genomes. The prevailing explanation to the pattern is that nucleosome occupancy favors higher G+C% sequences more than lower G+C% sequences. However, recent observation of a biased mutation spectrum caused by nucleosome occupancy suggests that the higher G+C% of nucleosomal DNA might be the evolutionary consequence of nucleosome occupancy. To distinguish the two explanations, we examined data from an in vitro nucleosome reconstitution experiment in which histones are incubated with yeast *Saccharomyces cerevisiae* and *Escherichia coli* genomic DNA, the former has been shaped by nucleosome structure while the latter has not. There is a strong positive correlation between nucleosome density and G+C% for the yeast DNA, an observation consistent with in vivo data, and such a pattern nearly vanishes for *E. coli* genomic DNA, suggesting that biased mutation, rather than biased occupancy, explains the most nucleosome-associated G+C% variation in eukaryotic genomes.

## Introduction

Most eukaryotic genomic DNA is packed into nucleosomes, the basic structural units of chromatin composed of approximately 147 base pairs (bp) of DNA wrapped around a histone octamer and connected by approximately 20–40 bp of unwrapped linker DNA. Because the nucleosome structure is crucial in regulating gene activity (Jiang and Pugh 2009), understanding factors that govern nucleosome position is thus highly desired. In fact, there is a long history in studying DNA sequences that favor or disfavor the occupancy of a nucleosome both in vivo and in vitro, with a general recognition of the DNA bending capacity as a key determinant (Zhurkin et al. 1979; Satchwell et al. 1986; Lowary and Widom 1998; Thastrom et al. 2004). Recent genome-wide studies further demonstrated the importance of DNA sequence in determining nucleosome positions(Yuan et al. 2005; Ioshikhes et al. 2006; Peckham et al. 2007; Liu et al. 2008; Kaplan et al. 2009), revealing or confirming a variety of sequence features that appear disproportionately in nucleosomal DNA relative to naked DNA; for instance, there are a few dinucleotide motifs that occur with a 10-bp periodicity throughout a nuclosome (Segal et al. 2006). Despite the seemingly apparent complexity regarding how DNA sequences affect nucleosome position, a model (Tillo and Hughes 2009) considering only local G+C% is able to generate a predicted genome-wide nucleosome profile highly similar to the true profile in the budding yeast *Saccharomyces cerevisiae*, with an overall performance comparable to a much more complicated model (Kaplan et al. 2009). Thus, difference of local G+C content appears to be the dominant sequence feature separating nucleosomal DNA from naked DNA, with relatively higher G+C% in the former than in the latter (Tillo and Hughes 2009).

The prevailing explanation to the above pattern is that nucleosome occupancy favors G+C richer DNA sequences than A+T richer sequences (Kaplan et al. 2009; Segal and Widom 2009b; Kenigsberg et al. 2010; Locke et al. 2010, 2013; Valouev et al. 2011). The critical experimental evidence behind such a view is that synthesized long ploy (A:T) tracts disfavor nucleosome occupancy in vitro (Iyer and Struhl 1995). However, another experimental observation that is often neglected is that synthesized long poly(G:C) tracts disfavor nucleosome occupancy to a similar extent (Iyer and Struhl 1995). Thus, while it is true that extremely A+T-rich DNA sequences, such as ploy(A:T) tracts, disfavor nucleosome occupancy (Segal and Widom 2009a), it does not predict that nucleosomes favor G+C richer DNA sequences than A+T richer sequences, which is particularly true for sequences with a moderate level of G+C content. In other words, the higher G+C% in nuclesomal DNA relative to naked DNA might be contributed by factors other than sequence preference of nucleosomes occupancy. Using comparative genomic analyses and an experimental evolution, we showed in a previous study that nucleosome occupancy is able to suppress specifically C->T, C->A, and A->T mutations, generating an equilibrium G+C% of approximately 50% in nucleosomal DNA and approximately 33% in naked DNA (Chen et al. 2012). This finding indicates that the relatively higher G+C% in nuclesomal DNA could be simply an evolutionary consequence of nucleosome occupancy.

The in vitro nucleosome reconstitution system comprising purified histones and genomic DNA eliminates confounding factors, thus able to capture the pure DNA features underlying nucleosome positioning. In such a system, the most commonly used genomic DNA is from eukaryotes (e.g., yeast). However, because the yeast genome has coevolved with nucleosomes over millions of years, it is difficult to identify the mechanism underlying the higher G+C% of nucleosomal sequences by using such DNA. A prokaryotic genome subject to no nucleosome-dependent mutation is then a good choice. Comparison of the in vitro nucleosome reconstruction profile between yeast and *Escherichia coli* may help us to find out the real reason behind the relative G+C richer of nucleosomal DNA-binding favors or evolutionary consequences of nucleosome occupancy.

## Materials and Methods

### Data Set

The nucleosome data from the in vitro nucleosome reconstitution experiment were downloaded from Genome Expression Omnibus (GEO; GSE15188). In that study, the yeast *S. cerevisiae* and *E. coli* genomic DNA were mixed in a 3:1 mass ratio and then incubated with purified histones by salt dialysis or by using a purified system containing recombinant *Drosophila melanogaster* NAP-1 and ACF as assistant factors; after reconstitution, the assembled chromatin was digested with MNase, and the mononucleosomal DNA was subsequently purified and sequenced by Illumina Genome Analyzer. As a control, the same *S. cerevisiae* and *E. coli* genomic DNA was sonicated into fragments of comparable size as to the mononucleosomal DNA and sequenced by the same platform.

We mapped the downloaded sequencing reads to reference genomes (*E. coli* K12 MG1655 ACCESSION: U00096 and *S. cerevisiae* Stanford Genome Database release R64-1-1) by using Burrows-Wheeler Aligner (BWA) (Li and Durbin 2009) with default settings. The resulting SAM files were subsequently processed by Picard (http://broadinstitute.github.io/picard/, last accessed March 25, 2015), to remove the polymerase chain reaction duplicates and nonuniquely mapped reads.

### Dinucleotides Distribution

One-pile data set of nucleosome core start-site positions was derived for both yeast and *E. coli* by taking all of the individual read start sites in the genome where one or more nucleosome core reads were detected. There are 1,460,637 and 191,597 unique start sites detected in yeast and *E. coli*, respectively, where the reconstruction experiment was done by using salt dialysis; and 3,141,959 and 1,380,939 start sites detected where assisted factors were supplied. By using the one-pile data set, we calculated dinucleotides distribution, for each position the result was smoothed by combining the result from two neighboring positions.

### Calculation of the Nucleosome Density

We computed genome-wide nucleosome density for each base pair by extending each read to 146 bp from its 5′-end. The genomes were then divided into consecutive fragments and the relative nucleosome density was calculated for each fragment. The relative nucleosome density of a fragment was calculated as the average nucleosome density of its all base pairs in the nucleosome reconstitution experiment divided by that of the control experiment using naked DNA. To facilitate comparison the relative nucleosome density was subsequently normalized by dividing the average density of all fragments from the corresponding genome, and all the fragments were binned according to their G+C% and comparison between different bins were performed. To eliminate the bias from sequencing and mapping a fragment was taken into consideration only when 90% region of the fragment was covered by sequenced reads of naked genomic DNA. Finally, there were approximately 230,000 and 90,000 fragments in yeast and *E. coli* genomes, respectively.

## Results and Discussion

We analyzed data of an in vitro nucleosome reconstitution experiment (Zhang et al. 2009), in which the yeast *S. cerevisae* genomic DNA and *E. coli* genomic DNA were pooled in a 3:1 mass ratio and then incubated with purified histones, followed by MNase digestion; the resulting mononucleosomal DNA was subject to Illumina sequencing. To control the potential bias due to the Illumina sequencing, the same naked genomic DNA was sonicated into fragments of comparable size to mononucleosomal DNA, and then sequenced on the same platform.

**Fig. 1.—:**
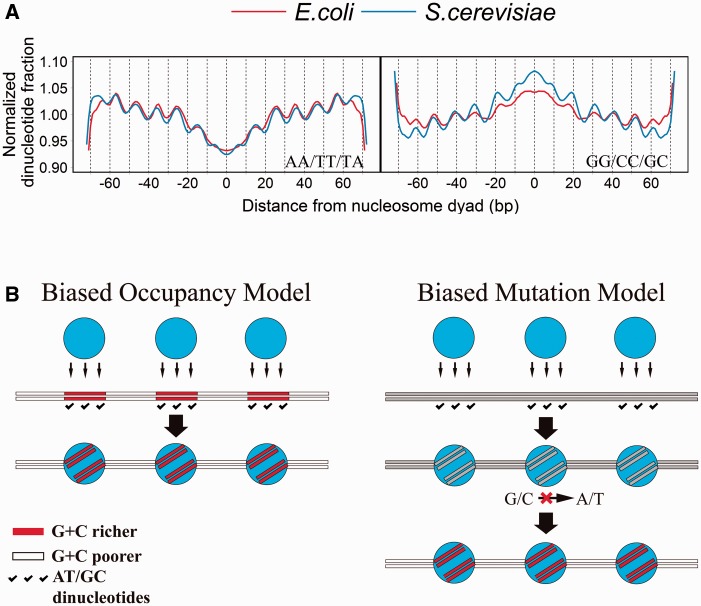
The two competing models for explaining the nucleosome-associated G+C% variation. (*a*) Dinucleotides distribution in nucleosomal sequences. Dinucleotides preference of AA/TT/TA and GG/CC/GC shows approximately 10-bp periodicity for both yeast *S. cerevisiae* and *E. coli* genomic DNA*.* Chromatin was assembled without assisted proteins supplied. (*b*) Two competing models for explaining the nucleosome-associated G+C% variation.

After mapping sequenced reads to yeast *S. cerevisiae* and *E. coli* genome, we derived unique nucleosome reads start set (one-pile data set) for both yeast and *E. coli.* By using this data, distribution of dinucleotides reveals oscillating approximately 10-base periodicity of AA/TT/TA and GG/CC/GC both existing in yeast and *E. coli*, especially when no assisted proteins were supplied (fig. 1*a* and supplementary fig. S1, Supplementary Material online)*.* This observation is highly consistent with previous study (Zhang et al. 2009) and indicates that intrinsic DNA features (e.g., dinucleotides preference) have a dominant role in nucleosome organization in both yeast and *E. coli* genome*.* These dinucleotides elements may generate intrinsically curved DNA and help establish the histone-DNA interface. Based on this result two competing models, here termed as biased occupancy model and biased mutation model (fig. 1*b*), both in principle are able to explain the observation of relatively higher G+C% in nuclesomal DNA. As we mentioned above the *E. coli* genome are evolutionarily neutral with respect to nucleosome formation, observation of increased nucleosome density in G+C-richer regions of *E. coli* genome would support the biased occupancy model, and the lack of such a pattern would reject the model. It is interesting to dissect their contribution to the nucleosome-associated G+C% variation within eukaryotic genomes.

**Fig. 2.—:**
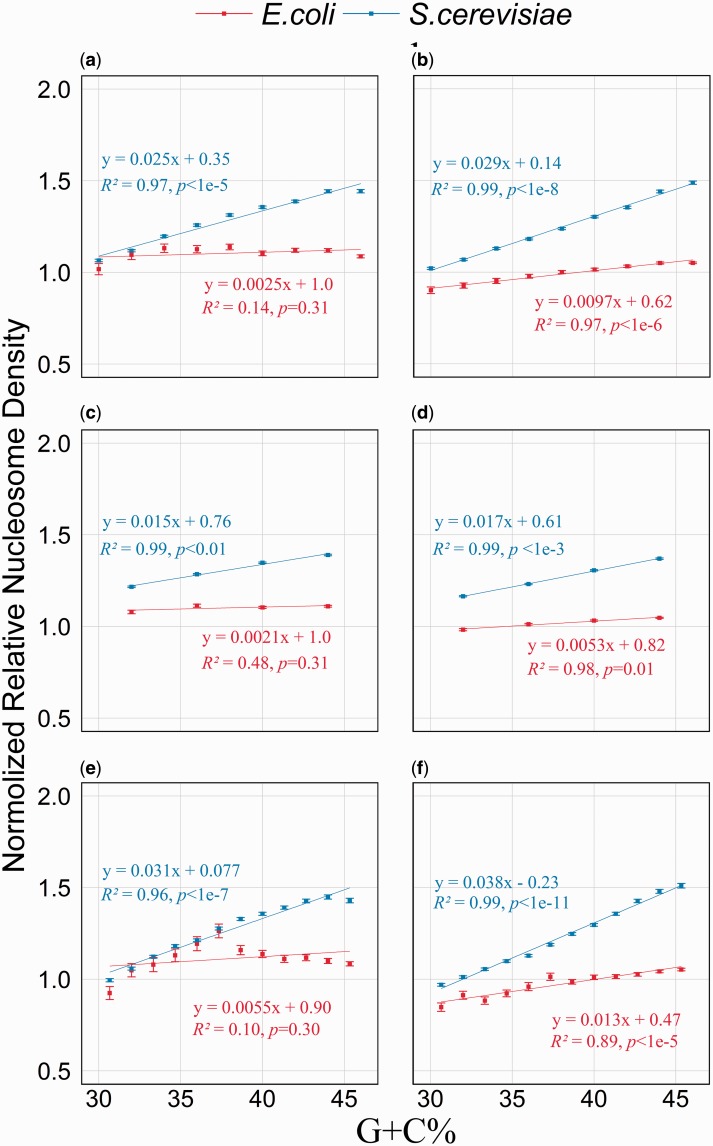
Significant increase of nucleosome density is observed accompany with the elevated G+C% for yeast genomic DNA, but not for *E. coli* genomic DNA. To make the two genomes comparable, the relative nucleosome density of a fragment is normalized by dividing the average nuclesome density of all fragments of the corresponding genome. For each bin, the mean relative nucleosome density and one standard error of the mean are shown (*y* axis), as a function of the G+C% of the bin (*x* axis). Data of in vitro nucleosome reconstitution with and without assisted factors are examined, with varied fragment sizes considered. (*a*) Without assisted factors; fragment size = 50 bp. (*b*) With assisted factors; fragment size = 50 bp. (*c*) Without assisted factors; fragment size = 25 bp. (*d*) With assisted factors; fragment size = 25 bp. (*e*) Without assisted factors; fragment size = 75 bp. (*f*) With assisted factors; fragment size = 75 bp.

Because it is difficult to assign regions with or without nucleosome occupancy, here we adopted a quantitative approach to examine the relationship between G+C% and nucleosome density. We divided both genome into 50 bp consecutive fragments, and calculated the relative nucleosome density of a fragment as the average nucleosome density of its 50 bp in the nucleosome reconstitution experiment divided by that of the control experiment using naked DNA. We binned fragments with similar G+C%, and compared between bins their relative nucleosome density. The G+C% of the yeast genomic regions examined here is approximately 38%, so we plotted for bins with G+C% from 30% to 46% their relationship between G+C% and nucleosome density (fig. 2). According to our results there is a strong positive correlation between nucleosome density and G+C% for the yeast DNA, with approximately 1.5-fold increase of the nucleosome density from the bin of 30% to the bin of 46% (fig. 2*a* and *b*). This result is in good agreement with previous studies (Segal and Widom 2009b; Tillo and Hughes 2009), demonstrating that our analytical strategy, despite being simple, works well. Interestingly, such a strong pattern cannot be observed for *E. coli* genomic DNA*.* Although there is either a slight or a strong positive correlation, the variation of nucleosome density is much smaller than that of yeast. For *E.coli* genomic DNA that without assisted proteins, the nucleosome densities of different G+C% are basically the same. When assisted proteins were supplied, despite there is a good positive correlation, the nucleosome density of the highest G+C% is only approximately 1.15-fold of that of the lowest G+C%. We further plotted the nucleosome density for all fragments from the whole G+C% range, and similar results were obtained. Although obvious positive correlation between nucleosome density and G+C% can be observed for yeast genomic DNA, such correlation became very slight for *E. coli* genomic DNA (supplementary fig. S2, Supplementary Material online)*.* To check whether fragment size affects the results, we divided, instead of 50 bp fragment, both genome into 25 or 75 bp fragment, and carried out the same analysis. We observed largely the same pattern (fig. 2*c*–*f*).

Therefore, our analyses suggest that the contribution of biased nucleosome occupancy is minimal, and most, if not all, of the nucleosome-associated G+C% variation should be explained by the biased mutation model. In the other words, although intrinsic DNA preference is one of the most important factors that can influence nucleosome positioning, the G+C richer is largely just the evolutionary consequence of nucleosome occupancy. Notably, we reached the same conclusion from a different angle in a previous study on genomic and nucleosome data of the Japanese killifish medaka (Chen et al. 2012). Nevertheless, although the biased mutation model may underlie the origin of some genomic regions with very low G+C% (e.g., promoters), these regions are now major disfavoring factors of nucleosome occupancy in eukaryotic genomes, highlighting the interplay of the two models in such regions.

The predominant role of the biased mutation model in shaping genomic regions with a moderate G+C%, such as coding sequences, has important implications for understanding protein evolution. There are a variety of basic issues, including mutation rate, mutation spectrum, codon usage, and amino acid usage that could have been affected by the nuclosome-associated biased mutations. Because it seems that many DNA-binding proteins can function like histones in suppressing C->T mutations (Warnecke et al. 2013), it should be cautious to interpret the so-called dual-used codons or “duons” recently proposed in human genes (Xing and He 2015), which are based on the observation of different synonymous codon choices in regions occupied by transcription factors(Stergachis et al. 2013). Another interesting fact is that such a genome-wide biased mutation pattern regulated in vivo by major DNA-binding proteins has recently been extended to bacteria (Warnecke et al. 2013).

## Supplementary Material

Supplementary figures S1 and S2 are available at *Genome Biology and Evolution* online (http://www.gbe.oxfordjournals.org/).

Supplementary Data
