# Supplementary material for: Mutation Bias, rather than Binding Preference, Underlies the Nucleosome-Associated G+C% Variation in Eukaryotes
Source: Genome Biol Evol. 2015 Mar 18;7(4):1033–8. doi: 10.1093/gbe/evv053 (PMC4419799; doi:10.1093/gbe/evv053)
Supplement: Supplementary Data [file supp_7_4_1033__index.html]

Mutation bias, rather than binding preference, underlies the nucleosome-associated G+C% variation in eukaryotes — Mutation Bias, rather than Binding Preference, Underlies the Nucleosome-Associated G+C% Variation in Eukaryotes — Supplementary Data 

# Mutation Bias, rather than Binding Preference, Underlies the Nucleosome-Associated G+C% Variation in Eukaryotes

## Supplementary Data

files

**Files in this Data Supplement:**

- Supplementary Data - docx file
