# Supplementary material for: Mutation Bias, rather than Binding Preference, Underlies the Nucleosome-Associated G+C% Variation in Eukaryotes
Source: Genome Biol Evol. 2015 Mar 18;7(4):1033–8. doi: 10.1093/gbe/evv053 (PMC4419799; doi:10.1093/gbe/evv053)
Supplement: Supplementary Data [file supp_evv053_SI-20150226.docx]

**Supplementary Information of “Mutation bias, rather than binding preference, underlies the nucleosome-associated G+C% variation in eukaryotes”**

Ke Xing and Xionglei He

State Key Laboratory of Biocontrol, School of Life Sciences, Sun Yat-sen University, Guangzhou, 510275, China

**Supplementary Information includes:**

Legends for Supplementary Figure 1 and 2

Supplementary Figure 1 and 2

**Legends for Supplementary Figures**

**Fig. S1 Dinucleotides distribution in nucleosomal sequences.** Dinucleotides preference of AA/TT/TA and GG/CC/GC shows ~10bp periodicity for both yeast *S.cerevisiae* and *E.coli* genomic DNA*.* Chromatin were assembled with assisted proteins supplied.

**Fig. S2 A positive correlation between nucleosome density and G+C% is observed for yeast genomic DNA, and such correlation become slight or even vanishes for *E.coli* genomic DNA.** To make the two genomes comparable, the relative nucleosome density of a fragment is normalized by dividing the average nucleosome density of all fragments of the corresponding genome.

**
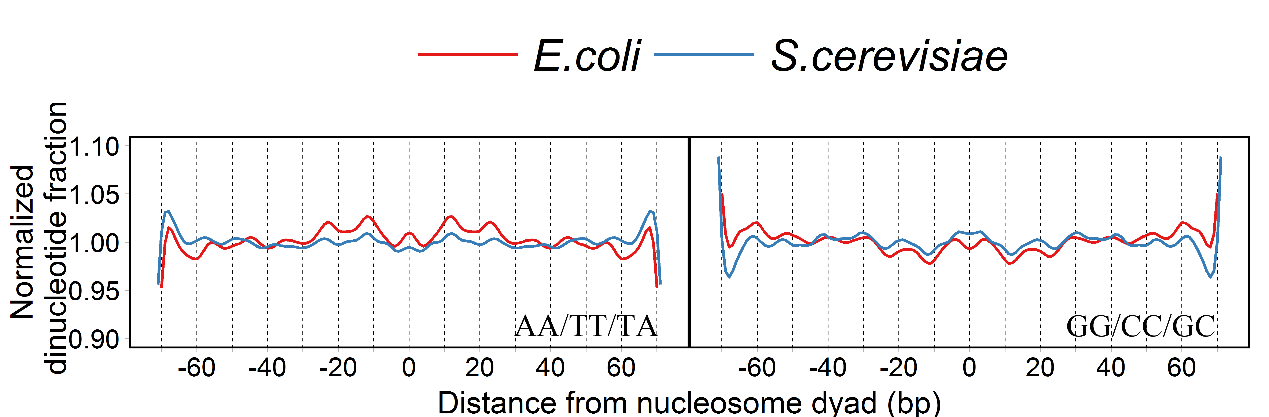
**

**Figure S1**

**
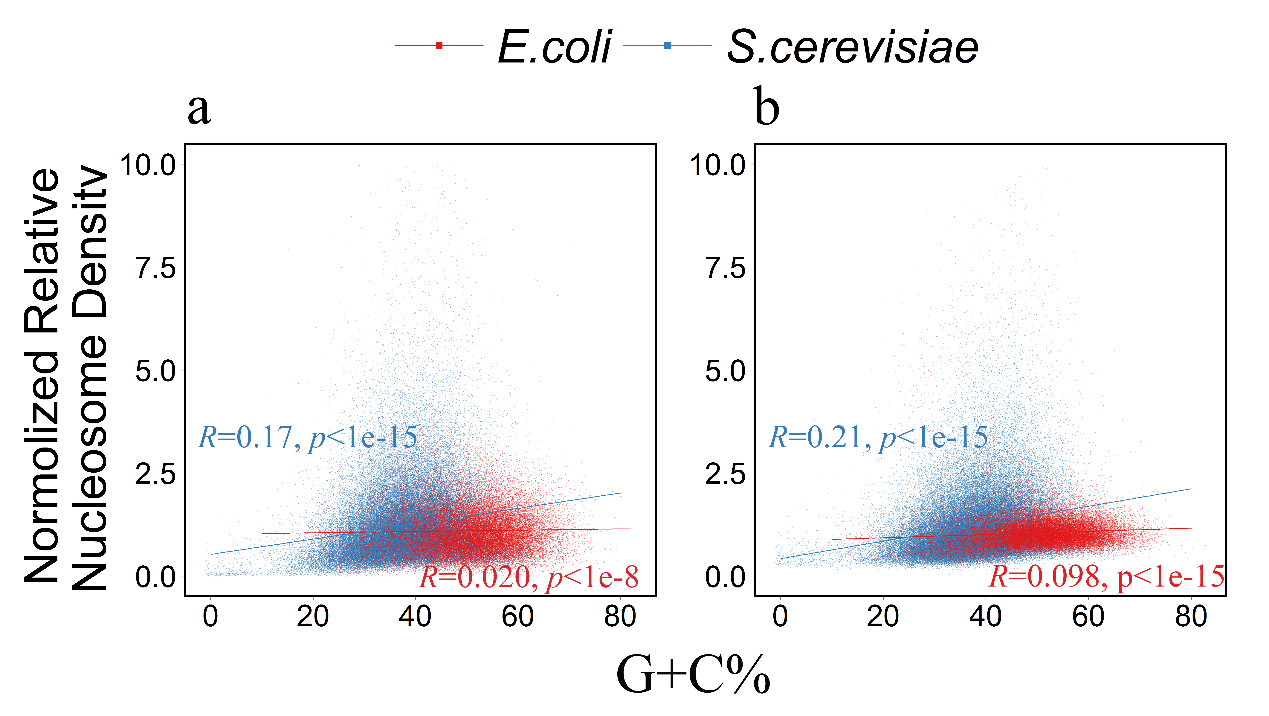
**

**Figure S2**
